# Whole-Genome Sequencing of Methicillin-Resistant *Staphylococcus aureus* Resistant to Fifth-Generation Cephalosporins Reveals Potential Non-*mecA* Mechanisms of Resistance

**DOI:** 10.1371/journal.pone.0149541

**Published:** 2016-02-18

**Authors:** Alexander L. Greninger, Som S. Chatterjee, Liana C. Chan, Stephanie M. Hamilton, Henry F. Chambers, Charles Y. Chiu

**Affiliations:** 1 Department of Laboratory Medicine, University of California San Francisco, San Francisco, California, United States of America; 2 UCSF-Abbott Viral Diagnostics and Discovery Center, San Francisco, California, United States of America; 3 Division of Infectious Diseases, Department of Medicine, San Francisco General Hospital, San Francisco, California, United States of America; Sidra Medical and Research Center, QATAR

## Abstract

Fifth-generation cephalosporins, ceftobiprole and ceftaroline, are promising drugs for treatment of bacterial infections from methicillin-resistant *Staphylococcus aureus* (MRSA). These antibiotics are able to bind native PBP2a, the penicillin-binding protein encoded by the *mecA* resistance determinant that mediates broad class resistance to nearly all other beta-lactam antibiotics, at clinically achievable concentrations. Mechanisms of resistance to ceftaroline based on *mecA* mutations have been previously described. Here we compare the genomes of 11 total parent-daughter strains of *Staphylococcus aureus* for which specific selection by serial passaging with ceftaroline or ceftobiprole was used to identify novel non-*mecA* mechanisms of resistance. All 5 ceftaroline-resistant strains, derived from 5 different parental strains, contained mutations directly upstream of the *pbp4* gene (coding for the PBP4 protein), including four with the same thymidine insertion located 377 nucleotides upstream of the promoter site. In 4 of 5 independent ceftaroline-driven selections, we also isolated mutations to the same residue (Asn138) in PBP4. In addition, mutations in additional candidate genes such as *ClpX* endopeptidase, *PP2C* protein phosphatase and transcription terminator *Rho*, previously undescribed in the context of resistance to ceftaroline or ceftobiprole, were detected in multiple selections. These genomic findings suggest that non-*mecA* mechanisms, while yet to be encountered in the clinical setting, may also be important in mediating resistance to 5th-generation cephalosporins.

## Introduction

Multi-drug resistant *Staphyloccocus aureus* (MRSA) is a ubiquitous problem in hospitals and in the community [1, 2]. The United States Centers for Disease Control and Prevention (CDC) estimates that there are over 75,000 MRSA infections annually [3], with the vast majority occurring in older persons in healthcare-associated settings. Ceftaroline is a fifth-generation cephalosporin with broad-spectrum activity against gram-negative and gram-positive bacteria, including MRSA [4], and is FDA-approved for skin and soft tissue infections, including those caused by MRSA [5, 6]. To date, a number of moderately ceftaroline-resistant strains (MIC 4 μg/ml) of MRSA have been described clinically. Moderate ceftaroline resistance has recently been shown to be particularly high among MRSA strains in China and Thailand. High-level ceftaroline resistance is rare, with only one clinical strain to date demonstrating resistance of MIC > 32 μg/ml [7–9]. Whole-genome and candidate gene sequencing have identified Y446N and E447K mutations in the *mecA / pbp2a* gene to be associated with high-level ceftaroline resistance [10, 11]. However, whether ceftaroline-associated resistance mutations exist outside of the *mecA / pbp2a* locus remains to be determined.

To discover potential *mecA*-independent resistance mechanisms, we sequenced the genomes of 7 total parent-daughter strains of *S*. *aureus* from which the *mecA* locus had been removed prior to passaging of ceftaroline or ceftobiprole *in vitro*. In addition, we sequenced and analyzed the full genome of a ceftaroline-resistant *mecA* positive strain (CRT) reported to exhibit non-*mecA* mediated ceftaroline resistance [10] in order to elucidate its mechanisms of resistance. Our data reveal a number of mutations in key genes within the *S*. *aureus* genome that are associated with the establishment of high-level resistance to ceftobiprole and ceftaroline.

## Materials and Methods

### Construction of strains

The parental strains for this study include Coln (a *mecA* positive strain) and Colnex and SF8300ex (strains from which the *mecA* gene has been excised) (Table 1). These parental strains were passaged daily 28 days, as previously described [12]. Briefly, 10 ml preparations of trypticase soy broth (TSB) containing various concentrations of antibiotic (ceftaroline or ceftobiprole) were inoculated at a 1:100 dilution with overnight cultures containing 10^9^ CFU ml^-1^. The drug concentration was doubled at each passage as tolerated until bacterial growth was observed in at least 128 μg ml^-1^ of the drug specified. At the end of passaging, a single clone was chosen randomly for further studies.

**Table 1:** Strains used in this study and mutations detected in penicillin binding proteins, gdpP and acrB genes.

Strains	MecA	Driver for selection	MIC to CFTR*	MIC to AMP**	MIC to NAF***	PBP1	PBP2	PBP3	*pbp4* promoter	PBP4	GdpP	AcrB
Coln^a^	(+)		1 S	16 R	128 R							
CRT^#^	(+)	CFTR	>64 R	256 R	>256 R		G631S		724602_724603insT (-377); 724500_724590del (-275)	N138K		
Colnex^b^^#^	(-)		<0.25 R	0.25 S	0.5 S							
CmTc^c^^#^	(-)	CFTR	>64 R	>256 R	>256 R		D156N		724602_724603insT (-377); 724624T>G (-399)	T201A; F241L	H443Y	
SF8300ex^d^^#^	(-)		0.25 S	0.25 S	0.5 S							
SRT^#^	(-)	CFTR	>64 R	>256 R	64 R				716955_716965del (-301)	N138K; H270L	Y306X	
SRB^#^	(-)	CFBP	4 R	4 S	8 S	H499R; E567K	Y437C; V445L; Q453R; M559I	W228X		E183V; F241R	T509A	
Sgap^e^	(-)		0.5 S	<0.25 S	0.5 S					E183A; F241R	N182K	I960V
SgapT^#^	(-)	CFTR	64 R	>256 R	>256 R	H499R			717031_717032insT (-377)	N138I; E183A; R200L; F241R	N182K	I960V
Sp^f^	(-)		0.25 S	0.25 S	1 S					E183A; F241R		
SpT^#^	(-)	CFTR	>64 R	>256 R	>256 R		G581D		716675del (-21); 717031_717032insT (-377)	N138I; E183V; T201A; F241R	N214del	

*CFTR = ceftaroline; CLSI breakpoints are ≤1 S, 2 I, ≥4 R (CLSI document M100-S23; ISBN 1-56238-865-7)

**AMP = ampicillin; CLSI breakpoints are ≤8 S, ≥16 R (CLSI document M100-S23; ISBN 1-56238-865-7)

***NAF = nafcillin; CLSI breakpoints are ≤8 S, ≥16 R (CLSI document M100-S23; ISBN 1-56238-865-7)

^#^Strains sequenced in this study

^a^Parental strain of CRT

^b^Col strain with mecA excised

^c^Colnex strain containing exogenous mecA plasmid (pYK20); mecA plasmid was evicted after ceftaroline selection (Chan L 2015 AAC)

^d^SF8300 strain with mecA excised, parental strain of SRT and SRB

^e^SF8300ex strain in which PBP4 (E183A, F241R), GdpP (N182K) and AcrB (I960V) mutations analogous to those in the CRB strain (Banerjee, et al. 2010 AAC) were introduced

^f^SF8300ex strain in which PBP4 (E183A, F241R) mutations analogous to those in the CRB strain (Banerjee, et al. 2010 AAC) were introduced

Abbreviations: MIC = minimal inhibitory concentration; R = resistant; S = susceptible, susc = susceptibility; CFTR = ceftaroline; CFBP = ceftobiprole.

The passaged daughter strains include (a) CRT, a ceftaroline-passaged derivative of the Coln strain [10], (b) CmTc, a strain in which a *mecA*-containing plasmid (*pYK20*) was re-introduced into Colnex before passaging in ceftaroline (CmTc) [10], and (c) SRT and SRB, two newly generated strains derived from passaging of SF8300ex in ceftaroline and ceftobiprole, respectively (Table 1). The mutant strain Sgap was created by introducing into SF8300ex select mutations from the *pbp4*, *acrB*, and *gdpP* genes that were identified via whole genome sequencing of a *S*. *aureus* strain selected for high-level ceftobiprole-resistance (CRB) [13]. Daughter strain SgapT was created by passaging the Sgap strain in ceftaroline. Finally, strain SpT was created by passaging in ceftaroline Sp, a mutant SF8300ex strain in which only *pbp4* mutations from strain CRB had been introduced.

Sgap and Sp were included in the study to determine if there were any differences in baseline ceftaroline resistance between a wild-type strain with only *pbp4* mutations (Sp) and a strain with mutations in *pbp4*, *gdpP*, and *acrB* (Sgap). Furthermore, passing of the Sp and Sgap strains were carried out in ceftaroline and subsequently genome sequenced to determine what changes were present in the resistant SpT and SgapT strains.

### Genomic DNA extraction

Bacterial cultures incubated in broth overnight (1 mL) were collected by centrifugation and re-suspended in 500 μL buffer containing 50 mM Tris-Cl, pH 8.0, 10 mM EDTA and 100 μg/mL RNase A. Bacterial suspensions were then transferred to Lysing matrix B column (MP Biomedicals) to lyse the bacteria. Bacterial lysates were incubated in ice for 5 min and centrifuged at 14,000*g* for 15 min. Supernatants containing the genomic DNA were transferred to a fresh tube, precipitated using ethanol and re-suspended in sterile water.

### Sequencing library preparation

One ng of genomic DNA was used as input for the Nextera XT kit (Illumina), followed by sample barcoding and amplification with 12 cycles of PCR. Libraries were quantified on the Bioanalyzer (Agilent Technologies) and combined in an equimolar mixture. Next-generation sequencing (NGS) libraries were sequenced on a single run on the Illumina MiSeq instrument (300 bp paired-end reads).

### Sequencing analysis

Reads were adapter- and quality-trimmed (Q30 cutoff; minimum length >20 nucleotides) using cutadapt [14], followed by re-pairing using pairfq [15]. Paired-end reads were mapped to the reference genomes of *Staphylococcus aureus* COL (NC_002951) or USA300 (CP000730) using Geneious v8.0 [16]. More than 97% of paired-end reads mapped to the reference genomes, with coverage ranging from 138X−287X. High-confidence variants were called using a minimum coverage of 25X and minimum variant frequency of 90%. All called variants were manually reviewed to correct large deletions that were erroneously called as SNPs. Remaining unmapped reads were *de novo* assembled to find genetic elements not present in the reference sequence. Of note, all USA300-based strains *de novo* assembled a ~3,100-bp contig with >99% identity to the *Staphylococcus aureus* SAP046B plasmid (GQ900404), which did not comprise part of the sequenced core genome of *Staphylococcus aureus* USA300 in the National Center for Biotechnology Information (NCBI) GenBank reference database.

### Accession Numbers

All genomic data from this study have been deposited publicly in NCBI under BioProject PRJNA293093.

## Results

### Four independent selections in ceftaroline result in similar *pbp4* coding and promoter mutations

To identify key genes associated with non-*mecA* mediated ceftaroline resistance in MRSA, we recovered the whole-genome sequences of 2 parental strains and 6 resistant daughter strains that had been passaged in either ceftaroline (n = 5) or ceftobiprole (n = 1) (Table 1). We first examined 6 genes that had been previously implicated in beta-lactam resistance in *Staphylococcus aureus* (Table 1), including *pbp1*, *pbp2*, *pbp3*, *pbp4*, *pbp4* promoter, *gdpP*, and *acrB* [10, 17, 18].

In the SRT strain, selected in ceftaroline directly from parental strain SF8300ex, 2 coding mutations (N138K and H270L) out of 7 resided in the *pbp4* gene. In addition, an 11-bp deletion at nucleotide position -301 base pairs (bp) was present in the promoter sequence directly upstream of the *pbp4* gene. A premature stop codon was also found in the *gdpP* gene of the SRT strain.

Similarly, mutations in *pbp4* were noted in the CRT strain, which was selected in ceftaroline from a different parental strain (Coln). Two of 8 total coding single nucleotide variants (SNVs) were found to be associated with *pbp4*, including a coding change in the PBP4 protein also seen in SRT (N138K) and a single nucleotide insertion of a thymidine at nucleotide position -377 bp of the *pbp4* promoter (S1 Table). The CRT strain also contained a 91 bp deletion in the *pbp4* promoter beginning at nucleotide -274 bp and a G631S mutation in *pbp2*. Outside of the *pbp* genes, two deleted regions 60 bp and 12 bp in length were presented in CRT, resulting in truncations in two metabolic proteins (TdcB and IIABC).

The SgapT strain, also selected in ceftaroline, contained 24 total coding mutations relative to its parental SF8300ex strain. Of these, three of 24 mutations were found in *pbp1* and *pbp4* genes coding for PBP1 and PBP4, respectively. Two of the coding mutations were detected in the *pbp4* gene (N138I and R200L) and a H499R mutation was found in *pbp1*. As in the CRT strain, a single nucleotide insertion of a thymidine at nucleotide position -377 bp was also present in the *pbp4* promoter.

The CmTc strain, derived from parental strain Colnex, had a total of 9 coding mutations. Four of these mutations were mapped to previously identified *mecA*-independent beta-lactam resistance genes [13, 19], including two coding mutations in *pbp4* (T201A, F241L), one mutation in *gdpP* (H443Y), and one mutation in *pbp2* (D156N). In addition, the CmTc strain had the same -377 bp thymidine insertion in the *pbp4* promoter as found in CRT and SgapT, as well as a T→G SNV that was not present in any of the other strains.

Comparison of the SpT strain to its grandparent SF8300ex strain demonstrated 24 coding SNVs in total. The SpT strain had four coding changes in the *pbp4* gene; however two of these changes (E183V, F241R) had been deliberately introduced into the parental strain, and the remaining two (N138I, T201A) were acquired SNVs during selection in ceftaroline. In addition, one coding mutation (G581D) was present in *pbp2* and a 3-bp deletion (N214del) was present in the *gdpP* gene. Importantly, as in SRT, CRT, SgapT, and CmTc, mutations in the *pbp4* promoter were present, including a single nucleotide insertion of a thymidine at -377 bp and a single nucleotide deletion of a thymidine at -21 bp.

**Fig 1:**
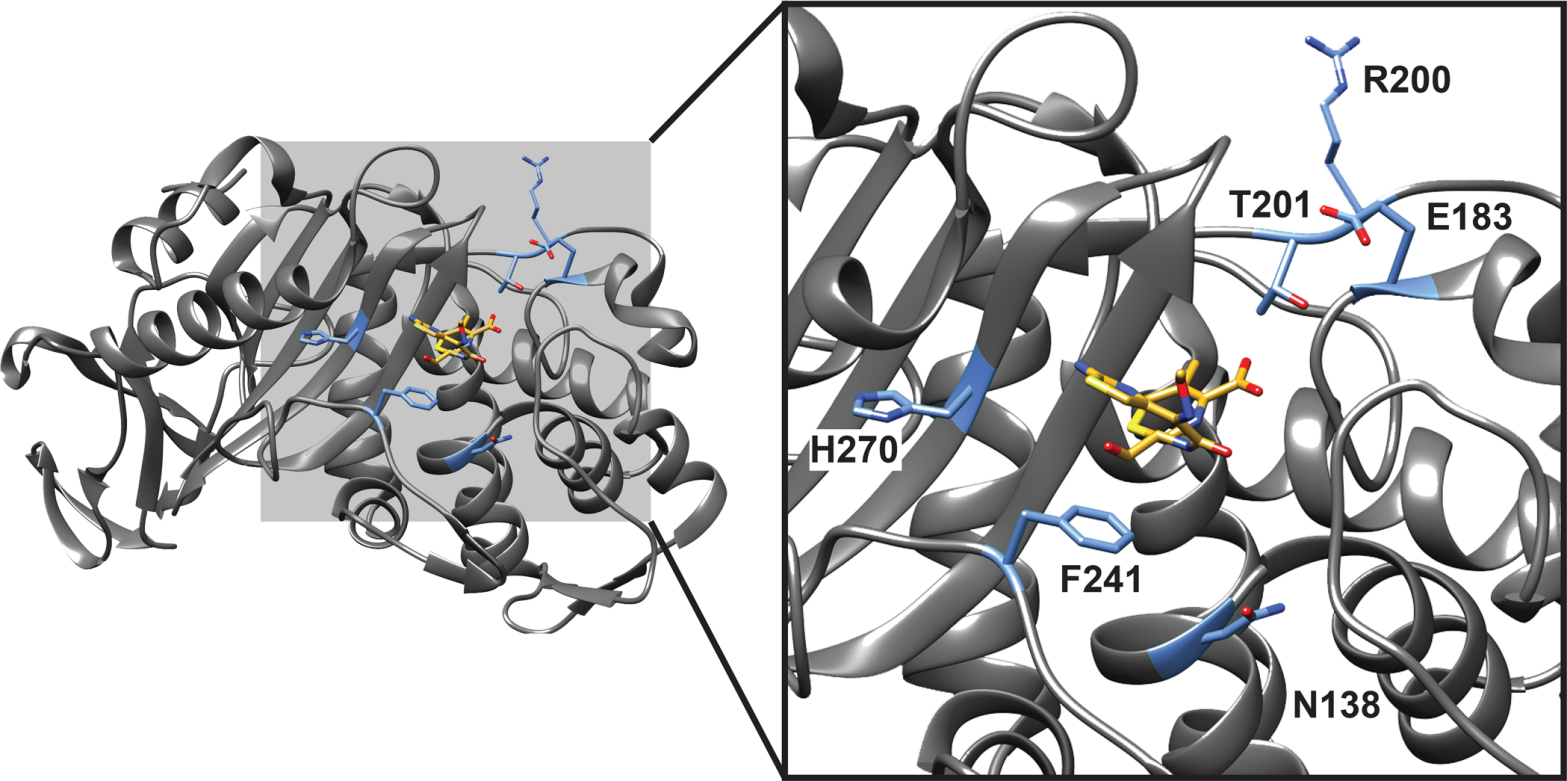
Mapping of pbp4 gene mutations to the crystal structure of the PBP4 protein of *Staphylococcus aureus* complexed with a cephalosporin antibiotic (cefaxotaxime). The left panel shows the entire complex, whereas the right panel shows a zoomed image of the cephalosporin binding pocket. The *Staphylococcus aureus* strain depicted in the crystal structure (PDB 3HUM) [30], Col, is the parental strain of the Colnex strain used in the current study (Table 1). Mutant residues in PBP4 identified by selection with ceftaroline or ceftobiprole are highlighted in blue. The ligand marked in yellow is cefotaxime.

Across the 5 passaged daughter strains selected in ceftaroline or ceftobiprole, a total of 6 mutations in the *pbp4* gene were identified (N138K, N138I, R200L, T201A, F241L, and H270L). Four of the 5 (80%) ceftaroline selections recovered a mutation to the Asn138 residue of *pbp4* (N138K or N138I). Mapping of the 6 *pbp4* mutations to the PBP4 crystal structure of *Staphylococcus aureus* Col strain in complex with cefotaxime (PDB 3HUM) revealed that all mutations were localized, as expected, to the cephalosporin-binding pocket (Fig 1). Importantly, all five of the ceftaroline selections also generated mutations to the *pbp4* promoter, with 4 out of 5 (80%) selections demonstrating an identical single nucleotide thymidine insertion at -377 bp.

### Recurrent mutations across multiple selections identify additional candidate genes for *mecA*-independent beta-lactam resistance

After characterizing mutations in the 6 genes known to be associated with beta-lactam resistance genes (Macheboeuf *et al*., 2006, Zapun *et al*., 2008), we next examined remaining coding mutations in other genes that were represented in at least 3 of the 6 ceftaroline or ceftobiprole daughter strains (S1 Table). Interestingly, only 3 additional genes met this criterion (Table 2). Strains CmTc, SgapT, and SpT contained coding mutations in transcription termination factor *Rho*. The S14 family endopeptidase *ClpX* and *PP2C* protein phosphatase genes were found mutated in the ceftobiprole-resistant SRB strain as well as the ceftaroline-resistant SRT and SgapT strains. Both SgapT and SRB strains included mutation of the same residue (glycine 169) in the *PP2C* protein phosphatase, a highly conserved MRSA gene on the basis of NCBI conserved domain searches (family cd00143) [20].

**Table 2:** Additional coding mutations present in at least three selections in this study. Mutated genes from Table 1 are not included.

CmTc	SRT	SRB	SgapT	SpT	Locus tag	Gene	Description
	Q31X	G169S	G169D		USA300HOU_1156	*pp2C*	possible PP2C protein phosphatase
	P323L	E354fsX358	V381E		USA300HOU_1666	*clpX*	S14 family endopeptidase ClpX
F241L			E356V	F83delinsX	USA300HOU_2109	*rho*	transcription termination factor Rho

## Discussion

In this study, we performed whole-genome sequencing of 9 total parent-daughter strains of *S*. *aureus* using an Illumina NGS platform to identify novel mutations associated with high-level, *mecA*-independent resistance to fifth-generation cephalosporins. Notably, all 5 ceftaroline selections generated mutations in the *pbp4* promoter, while 4 of 5 (80%) selections were associated with *pbp4* SNVs coding for a mutation in the ASN138 residue of PBP4. Previously undescribed mutations in additional candidate genes such as *ClpX* endopeptidase, *PP2C* protein phosphatase and termination transcription factor *Rho* were also detected in multiple screens. Our data suggest that mutations in the *pbp4* gene or its promoter are strongly associated with resistance to fifth-generation cephalosporins, although further biochemical characterization will be needed to confirm these findings. These results also illustrate the power of whole-genome sequencing of bacterial isolates to rapidly identify key genetic loci involved in resistance.

Previous work on ceftaroline resistance in *S*. *aureus* has suggested that PBP4 has poor affinity for ceftaroline [19, 21–23]. However, loss of the *pbp4* gene has been shown to confer a significant reduction in methicillin resistance, and it has been suggested this may be due to epistatic interactions of PBP4 with PBP2/2a that facilitate resistance to beta-lactam antibiotics [24]. Interestingly, a previously reported MRSA strain PVI [25], which is known to overexpress *pbp4*, is similar to the ceftaroline-resistant strains reported here in that the PVI strain also contains a deletion in the *pbp4* promoter (of size 90 bp) and an adenosine, rather than thymidine, insertion at position -377 bp. This suggests that the *pbp4* promoter mutations isolated in this study may also lead to PBP4 overexpression and promote resistance. Docking analysis of 19 beta-lactams with all available PBP structures identified Asn138 as part of the PBP4 active site (Fig 1) and, importantly, ceftaroline has higher affinity for PBP4 than the PBP1 and PBP2 proteins [26].

Our genomic sequencing also reveals mutations in new candidate genes such as *ClpX* endopeptidase, *Rho* transcription termination factor and *PP2C* protein phosphatase, and it is plausible that these mutations may contribute to high-level resistance to fifth-generation cephalosporins. Deletion of the *ClpX* gene in MRSA strain USA300 was previously shown to increase the level of resistance to beta-lactam antibiotics [27]. However, this is the first reported identification of mutations in *ClpX* occurring from selection with a beta-lactam antibiotic (ceftaroline / ceftobiprole). The *Rho* transcription termination factor has been shown to be associated with reduced susceptibility to beta-lactam antibiotics, with *Rho*-null mutants demonstrating reduced sensitivity to cephalosporins [28]. Finally, a PP2C-type protein phosphatase (IreP) has been implicated in a signal transduction system that controls cephalosporin resistance in *Enterococcus faecalis* [29].

## Supporting Information

S1 Table All mutations associated with strains analyzed in the current study. Tabs indicate the changes in a given strain relative to the comparison strain (“SRT-SF8300” refers to changes present in strain SRT when compared to strain SF8300).
(XLSX)
